# Proteomic analysis of extracellular vesicles from medullospheres reveals a role for iron in the cancer progression of medulloblastoma

**DOI:** 10.1186/s40591-015-0045-3

**Published:** 2015-10-13

**Authors:** Brigitte Bisaro, Giorgia Mandili, Alice Poli, Andrea Piolatto, Valentina Papa, Francesco Novelli, Giovanna Cenacchi, Marco Forni, Cristina Zanini

**Affiliations:** EuroClone S.p.A Research Laboratory, Molecular Biotechnology Centre (MBC), University of Turin, Turin, Italy; Centre for Experimental and Clinical Studies CERMS, Azienda Universitaria Ospedaliera Città della Salute e della Scienza Città di Torino, Turin, Italy; BioDigitalValley srl, Pont-Saint-Martin (AO), Turin, Italy; Department of Neuromotor and Biomedical Sciences, Alma Mater University of Bologna, Bologna, Italy; Department of Molecular Biotechnology and Heath Sciences, University of Turin, Turin, Italy

## Abstract

**Background:**

Medulloblastoma (MB) is the most common malignant childhood brain tumor with the propensity to disseminate at an early stage, and is associated with high morbidity. New treatment strategies are needed to improve cure rates and to reduce life-long cognitive and functional deficits associated with current therapies. Extracellular Vesicles (EVs) are important players in cell-to-cell communication in health and diseases. A clearer understanding of cell-to-cell communication in tumors can be achieved by studying EV secretion in medullospheres. This can reveal subtle modifications induced by the passage from adherent to non-adherent growth, as spheres may account for the adaptation of tumor cells to the mutated environment.

**Methods:**

Formation of medullospheres from MB cell lines stabilized in adherent conditions was obtained through culture conditioning based on low attachment flasks and specialized medium. EVs collected by ultracentrifugation, in adherent conditions and as spheres, were subjected to electron microscopy, NanoSight measurements and proteomics.

**Results:**

Interestingly, iron carrier proteins were only found in EVs shed by CSC-enriched tumor cell population of spheres. We used iron chelators when culturing MB cell lines as spheres. Iron chelators induced a decrease in number/size of spheres and in stem cell populations able to initiate in vitro spheres formation.

**Conclusions:**

This work suggests a not yet identified role of iron metabolism in MB progression and invasion and opens the possibility to use chelators as adjuvants in anti-tumoral chemotherapy.

## Background

Medulloblastoma (MB) is the most common malignant childhood brain tumor with a propensity to disseminate at an early stage [1]. Although multimodal treatments have improved survival rates for patients with MB [2], these tumors are associated with high morbidity [3]. In addition to the histological classification, an international consensus paper endorsed and refined a molecular classification into four groups based on meta-analysis of expression profiling [4]. This new stratification tool has not yet produced any clinical results in terms of new treatment strategies or improving the quality of life of survivors. The current standard of care for patients with MB involves surgery followed by craniospinal irradiation and chemotherapy. In infants and young children, radiation therapy is rarely used because of the risk of long-term neurocognitive deficits. Therefore, new treatment strategies are urgently needed to improve cure rates, to decrease neurotoxicity, and to reduce life-long cognitive and functional deficits associated with current therapies.

Recently, a role for Cancer Stem Cells (CSC) (also referred to as brain tumor-initiating cells) [5] in MB was proposed, which is a tumor with relevant molecular heterogeneity. We have previously described medullospheres (MBS) [6], obtained with a serum-free medium (enabling the formation and growth of spheres), in order to study CSC in vitro.

Furthermore, over the past decade, a new mode of intercellular communication has been described, namely the release of membrane vesicles known as Extracellular Vesicles (EVs)/Microvesicles/Exosomes [7]. EVs have been implicated in several physiological and pathophysiological processes, including tumor growth and progression [8]. There has been only one study reporting the role of EVs in MB [9], but relevant signaling molecules activated in MB have been studied in tumors of different histotypes [10–12]. Therefore, the study of EVs from MB cell lines enriched in Cancer Stem Cells is a promising approach aimed at gaining a greater insight into tumor cell adaptive modifications to the microenvironment, both in vitro and in vivo, and at finding new diagnostic tools and treatment strategies [9].

In the present study we identified, through a proteomic approach [13, 14], a set of proteins carried by EVs originating from MB cell lines cultured both in standard conditions of adhesion (MB) and as spheres (MBS). Interestingly, iron carrier proteins were only identified in EVs shed by CSC-enriched tumor cell populations. Iron depletion causes cell arrest between G1/S phases and leads to inhibition of cell proliferation and apoptosis suggesting that the use of iron chelators could be a novel approach in cancer treatment [15]. In fact, although iron chelation has been shown to protect against disease progression and/or limit iron accumulation in some rare neurological disorders and hemoglobinopathies [16], the role of iron in the progression of MB remains poorly understood. The use of iron chelators in our MBS culture resulted in a decrease in the number/size of spheres and caused a decrease in stem cell populations able to initiate the formation of in vitro spheres. In conclusion, this work clearly implicates iron metabolism in MB progression and invasion.

## Methods

### Cell culture, reagents and antibodies

DAOY cell line was purchased from ATCC (U.K.), UW228 and ONS-76 cell lines were kindly provided by Dr. Charles G. Eberhart (John Hopkins University, Baltimore, MD) with the agreement of Dr. Mike Bobola (University of Washington, Seattle, WA). MB cell lines DAOY, UW228 and ONS-76 were cultured at 37 °C, in 5 % CO_2_ as previously described [6]. Briefly, DAOY was cultured in MEM/EBSS supplemented with 10 % FBS, UW228 in DMEM/F12 10 % FBS and ONS-76 in RPMI supplemented with 10 % FBS.

Iron chelators: deferoxamine (DFO), 2-pyridylketone 4,4-dimethyl-3-thiosemicarbazone (Dp44mT) and Iron introduced through Ferric ammonium citrate (FAC) in cellular culture were purchased from Sigma-Aldrich (Milan, Italy). DFO, Dp44mT, and FAC were used at concentrations ranging from 5 to 100 μM, 0.1 to 5 μM, and 50 to 200 μM, respectively.

The stemness profile of the MBS were characterized by immunofluorescence staining: MBS were previously grown in low adherent conditions in order to allow sphere formation, and spheres were then stained with antibodies against β catenin (Cell Signaling Technology, Inc., Danvers, MA, USA) and SOX-2, FITC or PE secondary antibodies were purchased from Abcam (Cambridge, UK).

### Tumor sphere generation assay

In order to produce medullospheres (MBS) from DAOY, UW228 and ONS-76, cells were grown at confluence in adherent conditions, trypsinized, pelleted and plated (6 × 10^4^/ml) in ultra-low attachment T25 Flasks (Corning Inc., NY, USA) for a further 7 days in serum-free EUROMED CSC (EUROCLONE Spa, Pero, MI, Italy) medium. Culture conditioning based on low attachment flasks, specialized medium and formation of spheres enabled the expansion of the CSC population. Suspensions of spheres from all cell lines were collected and counted in 96-well plates by inverted microscopy (Olympus CKX41).

### Clonal sphere formation assay

Colony-forming efficiency and self-renewal ability were tested by limiting dilution.

DAOY, UW228 and ONS-76 adherent cells were grown and dissociated as described above and a single cell per well was plated in 150 μl of growth medium in a low adhesion 96-well culture plate. A volume of 25 μl of medium per well was added every 5 days. The number of clonal tumor spheres for each 96-well culture plate was evaluated after 14 days of culture.

### Isolation and analysis of EVs

EVs were obtained both from supernatants of MB adherent cell lines and from MBS cultured overnight in FBS-devoid medium. To obtain EVs, after centrifugation at 10,000 g for 20 min to remove debris, cell-free supernatants were centrifuged at 100,000 g (Beckman Coulter Optima L-90 K ultracentrifuge) for 1 h at 4 °C, washed in PBS and subjected to a second ultracentrifugation under the same conditions. EV pellets were then resuspended in 50 μL PBS or in RIPA lysis buffer. The protein content of EVs was quantified by the Bradford method (BioRad, Hercules, CA, USA). The presence and purity of EVs were verified by western blot analysis using antibodies against HSP70 and HSP90 from Abcam (Cambridge, UK), and CD63 purchased from Santa Cruz Biotechnology, Inc (Dallas, Texas, USA), which are specific markers of EVs . Moreover, analysis of the size distribution of EVs was performed using NanoSight NS300 with a 488 blue laser (NanoSight Ltd, Minton Park UK). Through the laser light source the particles in the sample are illuminated and the scattered light is captured by the camera and displayed on the connected computer running Nanoparticle Tracking Analysis (NTA). Using NTA, the particles are automatically tracked and sized based on Brownian motion and the diffusion coefficient (Dt). Results are displayed as a frequency size distribution graph and output to a spreadsheet.

### Transmission electron microscopy

EVs isolated after 100,000 g ultracentrifugation were resuspended in 50 μl PBS and a drop of 8 μl of resulting suspension was adhered to a Cu-Rh formvar-coated 200 mesh grid for 2 min. Absorbing paper was gently used to remove any excess of the suspension, by holding it close to the side of the grid, without making contact with the coated area. EVs were immediately fixed with 2.5 % glutaraldehyde in PBS for 1 min and rinsed with several drops of distilled water on the grid. EVs were then positively stained with 1 % uranyl acetate for 1 min. Subsequent observations were carried out by a ZEISS EM 109 Transmission Electron Microscope at 80 kV.

### Proteomic analysis

EVs from adherent DAOY, UW228 and ONS-76 cells and spheres were separated by SDS-PAGE on 4to 20 % Tris-Glycine Gels (Thermo Scientific) in triplicate; entire lanes of 1-DE gels were then cut out for mass spectrometry analysis.

MS analysis and database searches were performed as previously described [17]. Briefly, gel slices from Coomassie-stained gels were excised, and protein digestion was carried out with trypsin. MS analysis of peptides was performed using a MALDI-TOF spectrometer (MALDI micro MX; Waters, Milford, MA, USA) operating on reflectron mode. Samples were loaded onto the MALDI target using 1.5 μl of the tryptic digest mixed in a 1:1 ratio with a solution of α-cyanohydroxycinnamic acid (10 mg/ml) in 40 % v⁄v acetonitrile, 60 % v⁄v trifluoroacetic acid 0.1 %. Peak lists were generated by ProteinLynx (Waters, Milford, MA, USA) and data preparation was carried out using the following parameters: external calibration with lock mass using a mass of 2465.1989 Da for ACTH (adrenocorticotropic hormone), background subtract type adaptive combining all scans, and de-isotoping with a threshold of 1 %. The 25 most intense masses were used for database searches against the SWISSPROT database using the free search program MASCOT (http://www.matrixscience.com); the search settings allowed one missed cleavage with the trypsin enzyme selected, oxidation of methionine as a potential variable modification, a peptide tolerance of 100 ppm, taxon human. Hemopexin used in western blot analysis was a gift from E. Tolosano [18].

### Western blotting

Protein samples were extracted by RIPA (1 M Tris, 5 M NaCl, 1 % Triton, 1 % Na-deoxycolate, 1 % SDS) and separated on 4 to 20 % Tris-Glycine Gels. Western-blot analysis of adherent cells, MBS and EVs was performed as previously described [19], using the following antibodies against CD63, HSP90, HSP70, and Actin. Actin was purchased from Santa Cruz Biotechnology, and HPR mouse secondary antibodies from Cell Signaling Technology, Inc (Danvers, MA, USA).

### Statistical analysis

The results are representative of at least three independent experiments performed in triplicate and are expressed as the means ± SEM. Statistical analysis of the data was performed using the Student’s *t* test.

### Protein network

Proteins experimentally identified in both adherent cells and spheres were evaluated by means of a web platform tool known as ProteinQuest (PQ, BioDigitalValley srl, Pont-Saint-Martin, AO, Italy). PQ automatically retrieved all relevant biological information from PubMed abstracts and captions from free full text articles, US patents and Clinical Trials.

The image captions were extracted using the BFO Java library (http://bfo.com/) on the PDF version of the scientific papers [20].

The PQ database contains documents already tagged with biomedical dictionaries and ontologies such as proteins, drugs, cells, miRNAs, diseases, bioprocesses, clinical and biological techniques, etc. Each assignment of terms takes into account all the corresponding aliases, for disambiguation.

The biological relationships between identified terms and their translation into a relevant result can be explored by analyzing the co-occurrences for pairs of terms. The web application automatically generates graphs. In this study, the protein networks were generated by selecting the proteins that are differentially expressed in the experiments described above, already associated in the same papers (abstracts or captions). Networks connecting all of these proteins from the literature were visualized using Cytoscape [21], a popular software platform for network analysis, and further characterized by Bingo plugins [22] for Gene Ontology Analysis.

## Results

### Isolation of EVs and morphological characterization

**Fig. 1:**
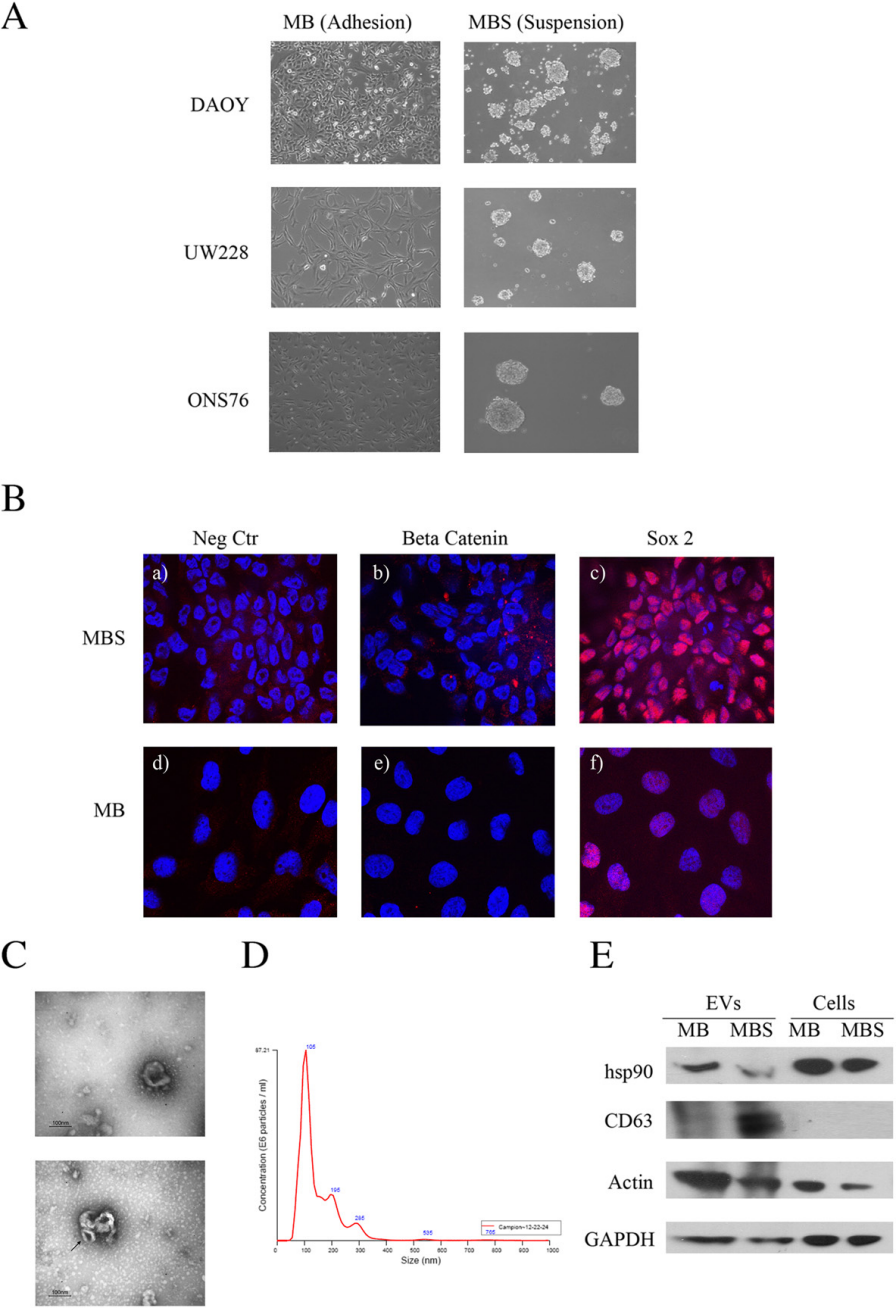
Purification of microvescicles from medulloblastoma cell lines and medulloblastoma spheres. **a** MB cell lines DAOY, UW228 and ONS-76 were cultured both in adhesion (MB) and as spheres (MBS). MBS were plated in ultra-low attachment flasks in EUROMED CSC serum-free medium (Euroclone code: ECM0894D). 4X magnification. **b** Representative image of immunofluorescence staining at 63X magnification of MB and MBS: (**a**, **d**) negative control, (**b**,**e**) β catenin (red), (c,f) Sox2 (red). **c** Electron microscopy representative images of exosomes from Medulloblastoma (MB) and Medulloblastoma spheres (MBS) from DAOY cells purified by ultracentrifugation. Note the cup-shaped morphology of vesicles (**a**). An aggregate of three exosomes (arrow) is clearly evident compared with smaller non cup-shaped vesicles in the background (**b**). **d** Nanoparticle tracking analysis (NTA) of MB and MBS EVs using the NanoSight instrument. Plot shows EV particle size distribution profiles and concentration measurements. The NanoSight instrument is based on a conventional optical microscope and uses a laser light source to illuminate nano-scale particles. **e** Western blot analysis of EV extracts from MB (adhesion), MBS (spheres) and of cells originating from EVs. Antibodies against HSP90, Actin and CD63 were used. CD63 is a specific marker of microvesicles (MVs). Actin was used as a loading control

Different medulloblastoma cell lines of (DAOY, UW228 and ONS-76) were cultured both in adherent conditions (MB) and as spheres (MBS) (Fig. 1a). As previously described [6], culture conditions of MB in low adhesion allowed formation of spheres enriched with CSC. To verify the stemness profile of MBS, immunofluorescence staining was performed using antibodies against β catenin and Sox2, specific markers closely linked to neuronal CSC. As shown in Fig. 1b, β catenin and Sox-2 were highly expressed in MBS compare to MB adherent cell lines. EVs from DAOY, UW228 and ONS-76 MB cell lines and MBS were then harvested by ultracentrifugation and characterized by ultrastructural morphology using electron microscopy (TEM) (Fig. 1c), NanoSight analysis (Fig. 1d) and western blotting (Fig. 1e). TEM analysis showed that the diameter of vesicles isolated through ultracentrifugation procedures ranged approximately from 50 to 120 nm and that these vesicles were surrounded by a lipid layer with a cup-shaped morphology. The vesicle surface appeared slightly wrinkled and they were present in both isolated and aggregated forms in the two culture conditions (adhesion and spheres). Moreover, smaller vesicles (<50 nm) not displaying the cup-shaped morphology, were sometimes observed and such vesicles were not considered as being exosomes. Finally, fragments of apoptotic cells, larger than exosomes, were occasionally observed. In conclusion, the great majority of EVs isolated through ultracentrifugation procedures were present as exosomes.

EVs were then analyzed by means of nanoparticle tracking analysis (NTA) using the NanoSight instrument (Fig. 1d) in order to visualize the size distribution of EVs. As shown in Fig. 1d, NanoSight analysis indicated that purified EVs had a diameter comprised between 50 to 120 nm and that the majority of particles displayed a 105 nm diameter, confirming the presence of exosomes. Finally, western blot analysis also confirmed the presence of EVs in exosome-form in the pellet obtained by the ultracentrifugation procedure, demonstrated by the positivity of the EV lysate for CD63 and HSP90, which are specific markers of exosomes (Fig. 1e).

### Identification of proteins carried by EVs

**Fig. 2:**
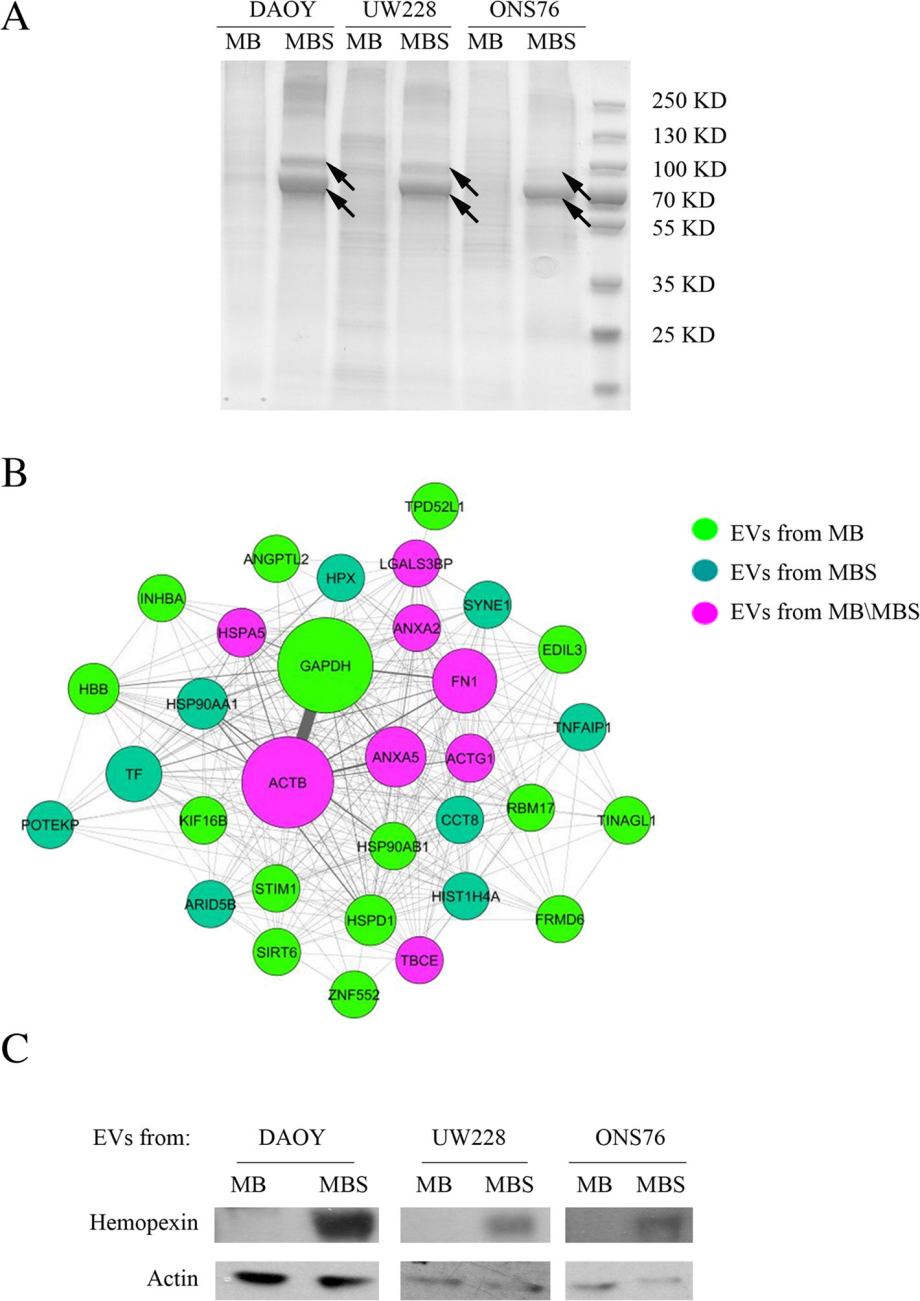
Proteomic analysis of EVs from MB, MBS and gene ontology analysis. **a** EVs from DAOY, UW228 and ONS-76 MB (adhesion culture) and MBS (spheres) were separated on SDS-PAGE. Coomassie-G bands were excised from the gels and destained. In-gel enzymatic digestions were performed before mass spectrometry analysis. **b** Representative network of the proteins (nodes) experimentally identified in adherent cells (purple), spheres (green) or both (magenta) and already described together in at least one paper (edges). The node size is proportional to the number of papers in which the proteins have been studied, while the edge width is proportional to the document number in which two proteins have been described together (data obtained by means of ProteinQuest). **c** Western blot analysis of EVs extracted from MB (adhesion), MBS (spheres) of DAOY, UW228 and ONS76 cells. Antibodies against Hemopexin were used. Actin was used as a loading control

To identify proteins carried by EVs, we performed three independent purification experiments of EVs derived from DAOY, UW228 and ONS-76, cultured both in adherent conditions (MB) and as spheres (MBS). The 1-DE pattern obtained after Coomassie staining of EV lysates revealed striking differences in the proteomic profiles of EVs from adherent cells (MB) vs. spheres (MBS), as indicated by the arrows (Fig. 2a). These differences were found in all the three cell lines of MBS EVs. The 74 most intense bands in the gels were cut and analyzed by mass spectrometry. Mass spectrometry analysis by MALDI-TOF identified 74 proteins: of these, 33 were unique proteins and the others were isoforms of the same protein. These 33 proteins identified (Table 1) were then analyzed using the ProteinQuest software in order to obtain a representative network in which EV proteins experimentally identified (nodes) in adherent cells (light green) and spheres (green) or in both (magenta) were linked together (edges) on the basis of the data from the literature (Fig. 2b). Indeed, the node size was proportional to the number of papers in which the proteins had been studied, while the edge width was proportional to the document number in which the two proteins were described together. The secretome of MB from adherent cells and spheres carries Annexin 2, Annexin 5, proteins with calcium channel activity and a potential role in cellular signal transduction, inflammation, growth and differentiation, galectin-3-binding proteins and 78 kDa glucose-regulated proteins, which play different roles such as monitoring protein transport through the cell.

**Table 1:** Maldi-Tof proteomic identification

	Abb.	Acc. N°		Full name	PM	p.l	M.pep	C %
shared_all	ACTG_HUMAN	P63261	ACTG1	Actin, cytoplasmic 2	41766	5,31	11	30
shared_all	GRP78_HUMAN	P11021	HSPA5	78 kDa glucose-regulated protein	72288	5,07	10	20
shared_all	ACTB HUMAN	Q96HG5	ACTB	Actin, cytoplasmic 1	41710	5,29	11	30
shared_all	ANXA2_HUMAN	P07355	ANXA2	Annexin A2	38580	7,57	13	29
shared_all	ANXA5_HUMAN	P08758	ANXA5	Annexin A5	35914	4,94	8	33
shared_all	TBCE_HUMAN	Q15813	TBCE	Tubulin-specific chaperone E	59309	6,32	7	16
shared_all	FINC_HUMAN	Q8IVI8	FN1	Fibronectin	262460	5,46	23	16
shared_all	LG3BP_HUMAN	Q08380	LGALS3BP	Galectin-3-binding protein	65289	5,13	11	23
shared_sph	HPX_HUMAN	P02790	HPX	Hemopexin	51643	6,55	10	26
shared_sph	TRFE_HUMAN	P02787	TF	Serotransferrin	77014	6,81	11	17
adh	CH60 HUMAN	P10809	HSPD1	60 kDa heat shock protein, mitochondrial	61016	5,2	7	20
adh	INHBA_HUMAN	P08476	INHBA	Inhibin beta A chain	47412	8,3	6	15
adh	STIM1_HUMAN	Q13586	STIM1	Stromal interaction molecule 1	77375	6,19	7	16
adh	FRMD6_HUMAN	Q96NE9	FRMD6	FERM domain-containing protein 6	71998	7,12	6	17
adh	SIR6_HUMAN	Q9NRC7	SIRT6	NAD-dependent protein deacetylase sirtuin-6	39094	9,31	6	19
adh	EDIL3_HUMAN	O43854	EDIL3	EGF-like repeat and discoidin I-like domain-containing protein 3	53730	7,08	8	20
adh	ANGL2_HUMAN	Q9UKU9	ANGPTL2	Angiopoietin-related protein 2	57068	7,23	7	19
adh	HBB_HUMAN	Q549N7	HBB	Hemoglobin subunit beta	15988	6,75	6	53
adh	HS90B HUMAN	P08238	HSP90AB1	Heat shock protein HSP 90-beta	83212	4,97	9	17
adh	G3P_HUMAN	P04406	GAPDH	Glyceraldehyde-3-phosphate dehydrogenase	36030	8,57	6	27
adh	ZN552_HUMAN	Q9H707	ZNF552	Zinc finger protein 552	46168	8,84	7	20
adh	KI16B_HUMAN,	Q8IYU0	KIF16B	Kinesin-like protein KIF16B	151918	5,86	12	10
adh	TINAL_HUMAN	Q9GZM7	TINAGL1	Tubulointerstitial nephritis antigen-like	52353	6,54	7	16
adh	SPF45_HUMAN	Q96I25	RBM17	Splicing factor 45	45162	5,76	6	17
adh	TPD53_HUMAN	Q5TDQ0	TPD52L1	Tumor protein D53	22435	5,46	5	19
sph	HS90A_HUMAN	P07900	HSP90AA1	Heat shock protein HSP 90-alpha	84607	4,94	8	15
sph	H4 UMAN	P62805	HIST1H4A	Histone H4	11360	11,36	5	51
sph	CHCH5 HUMAN	Q9BSY4	CHCHD5	Coiled-coil-helix-coiled-coil-helix domain-containing protein 5	12387	6,28	4	41
sph	SYNE1_HUMAN	Q9UJ06	SYNE1	Nesprin-1	1010456	5,37	18	3
sph	ACTBM_HUMAN	Q9BYX7	POTEKP	Putative beta-actin-like protein 3	41989	5,91	6	25
sph	TCPQ_HUMAN	P50990	CCT8	T-complex protein 1 subunit theta	59583	5.42	8	20
sph	ARI5B_HUMAN	Q9H786	ARID5B	AT-rich interactive domain-containing protein 5B	132292	8,89	8	10
sph	BACD2_HUMAN	Q13829	TNFAIP1	BTB/POZ domain-containing adapter for CUL3-mediated RhoA degradation protein 2	36181	8,26	6	28

33 proteins identified by Maldi-Tof and classified as follows: proteins present in EVs both from MB and MBS (shared-all), proteins expressed in all EVs from MBS (shared-sph) or only in adherent (adh) or spheres (sph) EVs

The gene ontology analysis of the EV protein network (Table 2) also highlighted a key role of other proteins through their bioprocesses, such as positive regulation of the nitric oxide biosynthetic process (*p* = 10^−5^), chaperone-mediated protein complex assembly (*p* = 10^−5^), heme metabolic process (*p* = 10^−5^), negative regulation of apoptosis (*p* = 10^−3^), iron ion homeostasis (*p* = 10^−3^) and positive regulation of angiogenesis (*p* = 10^−3^) involved in MB cell-to-cell communications.

**Table 2:** Gene ontology analysis of the EVs protein network

GO-ID	*P*-VALUE	Description of bioprocess	Proteins
45429	1.86E-05	positive regulation of nitric oxide biosynthetic process	HSP90AB1|HSP90AA1|HBB
20027	8.87E-05	heme metabolic process	IN H BA|HPX
51131	8.87E-05	chaperone-mediated protein complex assembly	HSP90AA1|HSPD1
43066	1.01E-03	negative regulation of apoptosis	TF| HSPD11 HSPA51ANXA51ANGPTL2
55072	2.56E-03	iron ion homeostasis	TF| HPX
45766	3.64E-03	positive regulation of angiogenesis	TF|ANGPTL2
ᅟ	ᅟ	ᅟ	ᅟ
GO-ID	*P*-VALUE	Description of molecular functions	Proteins
51082	3.30E-06	unfolded protein binding	HSP90AB1|HSP90AA1|CCT8|HSPD1|HSPA5
51087	3.16E-05	chaperone binding	TBCE|HSPD1|HSPA5
30235	4.14E-05	nitric-oxide synthase regulator activity	HSP90AB1|HSP90AA1
43498	8.17E-05	cell surface binding	TF|HSPD1|ANXA5
4859	2.71E-04	phospholipase inhibitor activity	ANXA5|ANXA2

The proteins listed in the table are categorized by their bioprocesses and molecular functions -GO Analysis

To further understand the role of these proteins, the molecular functions were also evaluated. In this way, the role of how these proteins exert cell-to-cell communications was defined: chaperone binding (*p* = 10^−5^), nitric-oxide synthase regulator activity (*p* = 10^−5^), cell surface binding (*p* = 10^−5^), and phospholipase inhibitor activity (*p* = 10^−4^) (Table 2).

Interestingly, iron carrier proteins (Hemopexin and Serotransferrin) were only identified in EVs shed by the CSC-enriched tumor cell population of all three MB cell lines analyzed. Therefore, to verify the robustness of the proteomic identifications, western blotting analysis was performed on EVs purified from MB and MBS. We confirmed that Hemopexin was highly expressed in DAOY, UW228, and ONS76 EVs originating from medullospheres compared to EVs coming from MB (Fig. 2c). Hemopexin was also expressed in MB cells and MBS which gave rise to EVs but at a very low level (data not shown), indicating a clear enrichment in the Hemopexin iron carrier protein in EVs from CSC. Therefore, on the basis of the proteomic results obtained in the EVs shed by MBS, we decided to investigate the influence of iron chelators on the formation of MB spheres.

### Role of iron in cancer progression

We used two distinct classes of iron chelators. The first was deferoxamine (DFO), a well-known iron chelator, widely used clinically for the treatment of iron overload in hemoglobinopathies, but impermeable to the cellular membrane. The second iron chelator was Dp44mT, a new compound able to pass through the cellular membrane. As a positive control we also added iron in the form of ferric ammonium citrate (FAC) to the culture medium of the spheres.

**Fig. 3:**
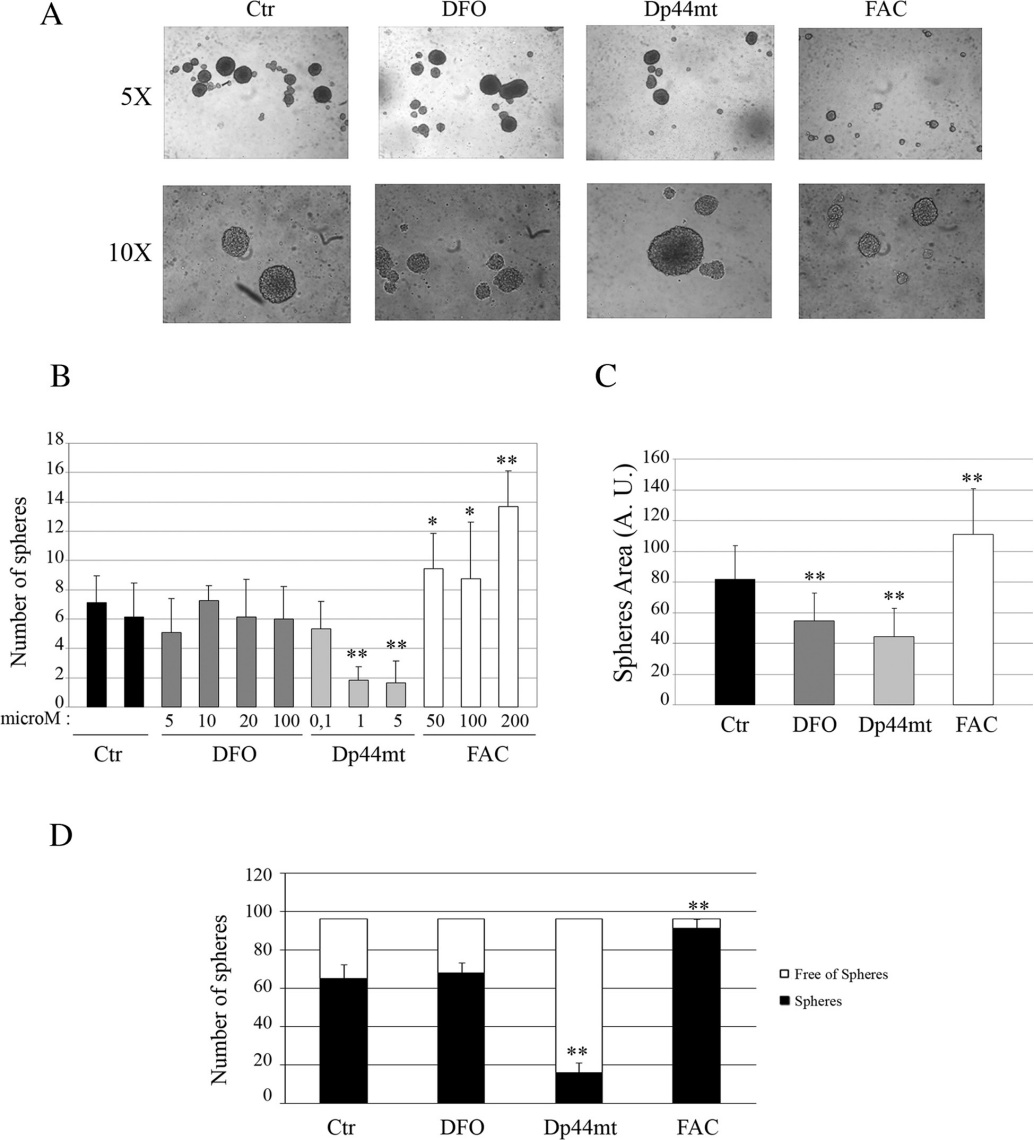
Iron chelation decreased UW228 medulloblastoma sphere formation and growth. **a** Representative image of UW228 spheres at 5X and 10X magnification in the presence of iron chelators in the culture medium (100 μM deferoxamine (DFO), 1 μM Dp44mT) or of iron (50 μM Ferric ammonium citrate). **b** Number of spheres (counted using a light microscope) obtained in a T75 low adhesion plate after 72 h of culture in the presence of different concentrations of DFO, Dp44mT or FAC (**p < 0.001). **c** Area of the spheres in arbitrary units (A.U) after 72 h of culture in the presence of different concentrations of DFO, Dp44mT or FAC (*p < 0.05). **d** Limited dilutions assay in which 1 cell was plated in each low adhesion culture 96 well-plate and after 14 days, the number of clonal tumor spheres formed in each well were counted in the presence of 100 μM deferoxamine (DFO), 1 μM Dp44mT or 50 μM of Ferric ammonium citrate (**p < 0.001)

**Fig. 4:**
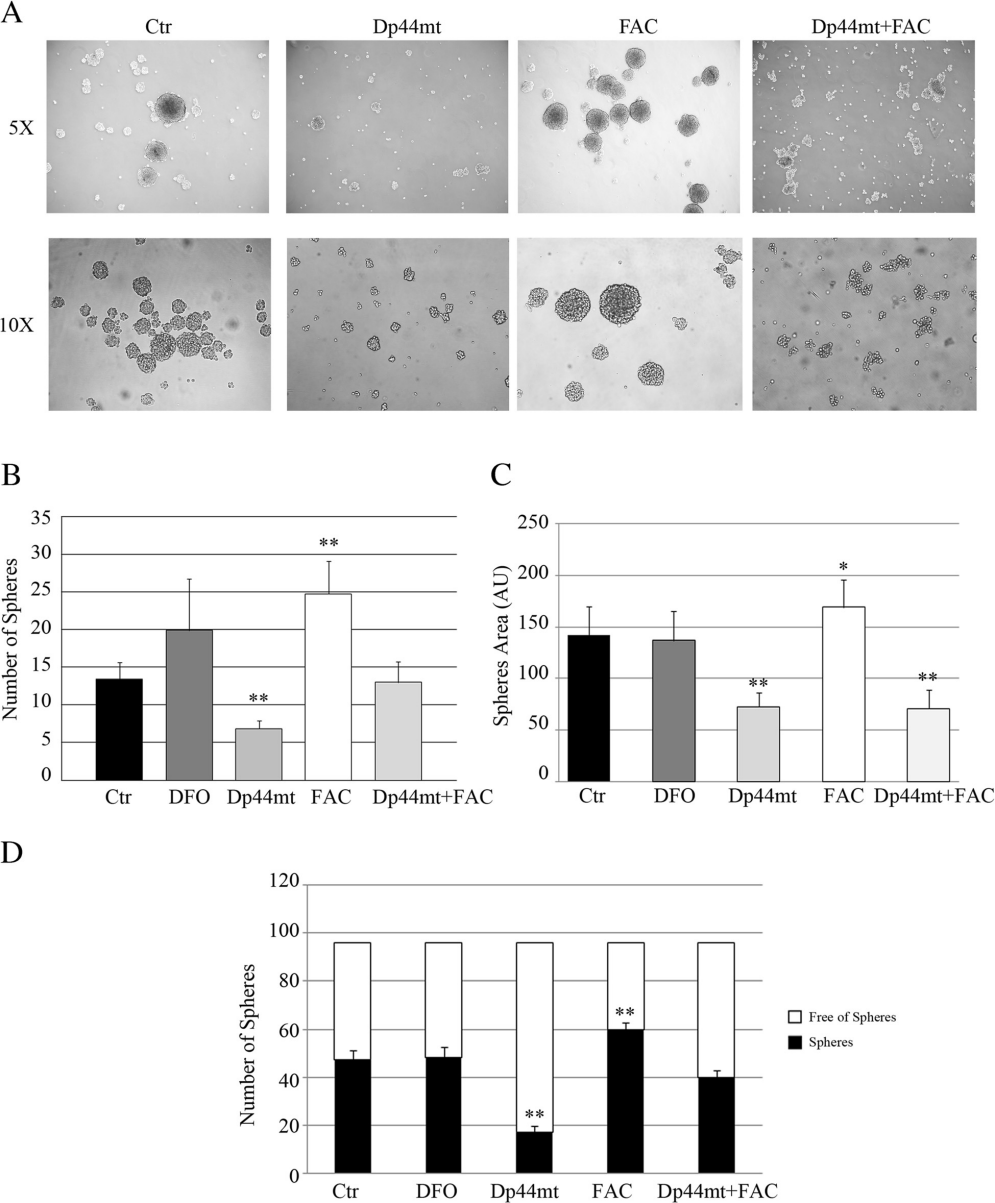
Iron chelation decreased DAOY Medulloblastoma sphere formation and growth. **a** Representative image of DAOY spheres at 5X and 10X magnification in the presence of iron chelators in the culture medium (100 μM deferoxamine (DFO), 1 μM Dp44mT) or of iron (50 μM Ferric ammonium citrate) or 1 μM Dp44mT + 50 μM FAC. **b** Count by light microscopy of number of spheres obtained in a T75 low adhesion plate after 72 h of culture in the presence of 100 μM DFO, 1 μM Dp44mT, 50 μM FAC or 1 μM Dp44mT + 50 μM FAC (*p < 0.05). **c** Area of the spheres in arbitrary units (A.U) obtained in a T75 low adhesion plate after 72 h of culture in the presence of 100 μM DFO, 1 μM Dp44mT, 50 μM FAC or 1 μM Dp44mT + 50 μM FAC (*p < 0.05). **d** Limited dilutions assay in which 1 cell was plated in each low adhesion culture 96 wells -plate and after 14 days, the number of clonal tumor spheres formed in each well were counted in the presence of 100 μM deferoxamine (DFO), 1 μM Dp44mT, 50 μM of Ferric ammonium citrate or 1 μM Dp44mT + 50 μM FAC (*p < 0.05)

Results obtained on UW228 MB cell lines revealed that using a concentration of 1 μM of the Dp44mT iron chelator on MBS cultures induced a 3-fold decrease in the number of spheres and a 2-fold decrease in sphere size compared to control spheres (Fig. 3a, b, c). DFO, which is not permeable to cellular membranes did not have any effect on the spheres. Moreover, adding iron to the cell culture, by means of 50 μM FAC, induced a 2-fold increase in the number and a 1.3-fold increase in the size of spheres compared to controls (Fig. 3a, b, c). Finally, MB cell lines were cloned by limiting dilution of dissociated cells by plating one single cell per well into 96 well-culture plates in the presence of 100 μM DFO, 1 μM Dp44mT or 50 μM FAC. Dp44mT induced a 3-fold decrease in the number of spheres formed by single cells and, conversely, the presence of iron (in the form of FAC), resulted in a 3-fold increase in the number of spheres compared to control cells (Fig. 3d). These data indicate that a population of sphere-generating cells with self-renewal ability is enriched in culture medium containing iron, and that iron chelation seems to be able to decrease this self-renewal ability. Similar experiments were performed on the DAOY cell line using fixed concentrations of iron chelators and FAC and adding Dp44mT and FAC to the medium for sphere culture (Fig. 4a). In DAOY MBS formations, the presence of Dp44mT induced a 2-fold decrease in the number of spheres formed compared to controls. Conversely, the presence of iron in the culture medium increased the number of spheres by 2-fold. The co-presence of Dp44mT and FAC restored the number of spheres to control levels. Again, the DFO chelator did not show any effect on sphere formation (Fig. 4b). Dp44mT also induced a decrease in sphere size (Fig. 4c) and in stemness ability (Fig. 4d), and the presence of iron was shown to favor the increase of the area of the spheres (Fig. 4c) and of the stemness capacity (Fig. 4d). Finally, the co-presence of Dp44mT and FAC decreased the area of the spheres to that observed with the use of Dp44mT alone (Fig. 4c), but restored the population of sphere-generating cells to control levels (Fig. 4d). These data indicate that iron depletion and/or the presence of iron can modulate the tumorigenic properties of MB cells in vitro, affecting stem cell populations and cell-renewal abilities.

## Discussion

A great deal of interest has been focused on exosomes and microvescicles from mesenchymal stem cells [23] and tumor cells as important means of intercellular communication [24]. Depending on the type of cells of origin and microenvironment investigated, EVs have been demonstrated to influence several different aspects of regulation, ranging from inflammation and repair to immunity, invasion and metastasis. To date, no completely unique protein marker for exosomes has been identified in order to verify that the sample of EVs contained purified exosomes and no cellular debris. As a result, a combination of methods was required to characterize the EVs, including determination of size and morphology of the EVs by electron microscopy, NanoSight analysis and western blotting [23–26]. In addition, the purity of the sample can be determined by electron microscopy, and this method can provide an overview of the level of contamination of the sample; for example, with larger vesicles, such as microparticles, apoptotic bodies or cell debris could be present in the cell cultures, indicating poor condition. When isolating EVs by ultracentrifugation, smaller vesicles (<50 nm), not displaying the cup-shaped morphology, can sometimes be present which significance is to be deepened. NanoSight analysis gave indications of EV size distribution and concentrations. NanoSight could indicate the diameter dimension of the majority of EVs (predominant peak proportional to concentration) and could also indicate the presence or absence of aggregates (peaks exceeding 200 nm). Finally, it was useful to perform a molecular analysis of protein markers known to be expressed by exosomes through western blotting analysis, showing the presence of specific markers such as CD63 and HSP90. The combination of these three analytic methods can demonstrate the presence of exosomes in the purified preparation of EVs.

The increasing interest in exosomes and microvesicles in organic fluids and exhausted culture media of different cell lines has opened new scenarios, and the role of EVs as intercellular messengers has been widely investigated in different physiological and pathological states. In parallel, great efforts have been directed towards finding new methods for early diagnosis of malignant tumors of infancy and childhood, as well as to find innovative and more effective therapeutic tools and strategies. EVs may also reflect metabolic conditions of tumor cells in culture and in Cancer Stem Cells. EVs shed by tumors have been investigated as important factors as they act on the tumor microenvironment and have been designated TEX (Tumor-associated Exosomes) recognizing their role in the modification of tumor-associated stroma from an inflammatory phenotype towards a reparative one [27]. The most specific bioprocesses characterized in EV proteins revealed that, in MB tumors, EV communication sustains stemness, survival and proliferation. As culture in suspension, or rather spheres, in our case “medullospheres”, induced an enrichment of Cancer Stem Cells, the modification of the protein repertoire found in EVs purified from medullospheres could most likely reflect an increased stemness compared to the same cells grown in adherence. The presence of medulloblastoma exosomes from cell lines D283MED, DAOY, and UW228, has been previously described [9].

Comparing our proteomic analysis with previous proteomic works on medulloblastoma [9] and brain tumor [28] exosomes, many proteins are shared, as expected: ACTB, G3P, GRP78, ANXA2, TRFE, CH60, HBB, HSP90. Other identified proteins are not exactly the same, but belong to the same functional class: TBCE (tubulin polymerization-promoting protein family member 2), ZN552 (other zinc finger proteins), KI16B (kinesin family member 16B), H4 (other histone proteins), CH60. The presence of proteins related to iron metabolism, namely Hemopexin, a Heme-binding protein with cytoprotective activity from free heme toxicity, and serum transferrin was an unexpected finding limited to EVs from medullospheres [15]. These proteins are iron carrier proteins, suggesting a possible implication of iron and heme metabolic processes, namely iron ion homeostasis (*p* = 10^−3^) and heme-related metabolic processes (*p* = 10^−5^) in MB progression and invasion (Table 2). The importance of iron for cells is related to the key role of Fe-containing proteins involved in oxygen sensing, energy, metabolism, respiration, folate metabolism and DNA synthesis (RR = ribonucleotide reductase). Without iron, G1 – S progression is hampered for normal and tumoral cells [29].

Iron has been recently described as an essential element in tumor cell proliferation, supporting energy and structural requirements of neoplastic cells. The role of iron in tumors has been intensively studied in all phases of tumorigenesis, and a specific influence of iron on tumor cell growth and tumor progression has been revealed in many human cancers [30, 31]. Most studies on iron and cancer were performed on epithelial or hematopoietic tumors and in vivo experiments; a further difficulty is to distinguish between the lowered iron concentration in tissues and organs and the direct effect of chelation on tumor cells. Although DFO and Dp44mT bind trivalent Fe, they have different actions. DFO is a hexadentate chelator of Fe^3+^ with high affinity, forming a 1:1 complex with iron and limited cell membrane permeability. Dp44mT forms a complex with ferric iron in a 2:1 ratio, with easy access into the cells. Furthermore, Dp44mT forms redox-active iron and copper complexes inside the cell, inducing the generation of ROS and relevant cytotoxicity via a Fenton type chemical reaction (Fe^2+^ H_2_O_2_ -- reactive intermediates – Fe ^3+^ + ^.^OH + OH^−^) [32].

In vivo studies have demonstrated that Dp44mT and related molecules do not lower iron concentration in tissues and biological fluids in the same way that DFO does. These data are in line with a prominent intracellular accessibility of Dp44mT compared to Deferoxamine.

An interesting interpretation of the results may be the fact that, although the main target of the compounds is iron chelation, other ions (such as Cu, as well as non-metallic ions) are bound by chelators.

In fact new iron chelators, such as Dp44mT, show anti-tumoral activity unrelated to iron deprivation but which interfere with relevant metabolic pathways [33].

More than half of human cancers express multidrug-resistant P-glycoprotein (Pgp) and Pgp-dependent lysosomal damage and cytotoxicity has been demonstrated for Dp44mT and related to Copper chelation. Several other pathways, in different tumors, are targeted by these new chelators, and the related anti-tumoral activity does not involve Fe sequestration as a primary cause.

Our results obtained on iron chelation demonstrated that only the permeable iron chelator Dp44mT is able to decrease sphere formation and stemness ability, highlighting a crucial role of intracellular iron in supporting tumor progression by EV communication. Moreover, the co-presence of iron and Dp44mT in medullospheres restored the number of spheres and cancer stem cells control levels, emphasizing the role of iron in cancer stem cell formation and enrichment.

## Conclusions

In conclusion, our findings on MB cell lines modulating iron-related proteins in different growth conditions are of interest and create possible scenarios linking iron metabolism to hypoxia, and could be potentially related to increased cell proliferation, tumor aggressivity and stemness. The influence of iron on the cellular division rate has been studied in different tumors and has opened the possibility to use chelators as adjuvants in antitumoral chemotherapy.
